# Characterization of DNA Binding Sites of RokB, a ROK-Family Regulator from *Streptomyces coelicolor* Reveals the RokB Regulon

**DOI:** 10.1371/journal.pone.0153249

**Published:** 2016-05-04

**Authors:** Paulina Bekiesch, Karl Forchhammer, Alexander Kristian Apel

**Affiliations:** 1 Pharmaceutical Biology, Pharmaceutical Institute, Eberhard-Karls-Universität Tübingen, Auf der Morgenstelle 8, 72076, Tübingen, Germany; 2 German Centre for Infection Research (DZIF), Partner site Tübingen, 72076, Tübingen, Germany; 3 Microbiology/Department of Organismic Interactions, Interfaculty Institute of Microbiology and Infection, Eberhard-Karls-Universität Tübingen, Auf der Morgenstelle 28, 72076, Tübingen, Germany; University Paris South, FRANCE

## Abstract

ROK-family proteins have been described to act either as sugar kinases or as transcriptional regulators. Few ROK-family regulators have been characterized so far and most of them are involved in carbon catabolite repression. RokB (Sco6115) has originally been identified in a DNA-affinity capturing approach as a possible regulator of the heterologously expressed novobiocin biosynthetic gene cluster in *Streptomyces coelicolor* M512. Interestingly, both, the *rokB* deletion mutants as well as its overexpressing mutants showed significantly reduced novobiocin production in the host strain *S*.*coelicolor* M512. We identified the DNA-binding site for RokB in the promoter region of the novobiocin biosynthetic genes *novH-novW*. It overlaps with the *novH* start codon which may explain the reduction of novobiocin production caused by overexpression of *rokB*. Bioinformatic screening coupled with surface plasmon resonance based interaction studies resulted in the discovery of five RokB binding sites within the genome of *S*. *coelicolor*. Using the genomic binding sites, a consensus motif for RokB was calculated, which differs slightly from previously determined binding motifs for ROK-family regulators. The annotations of the possible members of the so defined RokB regulon gave hints that RokB might be involved in amino acid metabolism and transport. This hypothesis was supported by feeding experiments with casamino acids and L-tyrosine, which could also explain the reduced novobiocin production in the deletion mutants.

## Introduction

ROK regulators are a group of proteins belonging to the ROK protein family [1]. This family is characterized by matching pfam entry PF00480 [2]. The pfam database lists 5087 sequences with PF00480 entry from 1397 species (data collected 18.12.2015, pfam.xfam.org/family/PF00480). ROK-family proteins are widespread among bacteria, but there are as well members among eukaryotes and archaea. Corresponding to its name ROK (**r**epressor, **o**pen reading frame, **k**inase) the family consists of three groups of proteins. The catalytically active sugar kinases with an N-terminal ATP-binding motif are well studied, regarding tertiary structure [3–5], substrate specificity [6, 7] and biochemistry [8, 9]. Recently, some ROK-family regulators containing an N-terminal helix-turn-helix DNA-binding motif have been investigated [10–12]. In 2005, the first structure of a ROK-family regulator, the global regulator of sugar metabolism Mlc from *Escherichia coli*, was solved [13]. Still many ROK-family proteins remain uncharacterized.

Most of the studied ROK-family regulators are involved in carbon catabolite repression and sugar metabolism. CsnR from *Streptomyces lividans* for example is a repressor of the chitosanase gene *csnA* [14], RafR from *Bifidobacterium breve* UCC2003 regulates the transcription of a raffinose utilization gene cluster [15] and Mlc and NagC control uptake and use of glucose and N-acetylglucosamine in *E*. *coli* [16, 17]. For the phylum *Thermotogae* a number of ROK-family regulators involved in sugar metabolism have been investigated regarding their DNA-binding site and substrate specificity [18]. For most ROK-family regulators, DNA-binding was not dependent on the presence of effector molecules, as e.g. in the case of RokA from *Streptococcus pneumoniae* D39 [11] or RafR from *B*. *breve* UCC2003 [15]. However, dependence on effectors to achieve binding was shown for other Rok regulators, e.g. for CysR in *Corynebacterium glutamicum* ATCC 13032 [12]. In addition, weakening of the binding in presence of effectors could also be observed [14, 18].

Streptomycetes, high G+C content Gram-positive bacteria, undergo a complex morphological differentiation and are able to produce a wide variety of secondary metabolites such as antibiotics, immunosuppressants, insecticides and antitumor agents [19, 20]. The onset of secondary metabolite production is coupled to the end of vegetative growth due to starvation or environmental changes. This developmental program requires complex changes in global gene expression [21]. Knowledge of the complex regulatory cascades and networks can provide us with the potential to awake cryptic gene clusters or increase production of known secondary metabolites [22].

Although ROK-family regulators, with their possible regulatory effect on primary and secondary metabolism, represent good candidates for manipulation of regulatory networks, only few ROK-family regulators from *Streptomyces* have been investigated until now. For CsnR from *Streptomyces lividans* TK24 a regulatory function on the *csn* genes was proven. A binding site (5′-CCTCTTCTGGTAGGAAACTTTCCTATCAGT-3′) was determined and products of chitosan degradation seem to weaken CsnR binding to its target DNA [14]. The ROK-family regulator Rok7B7 (Sco6008) from *S*. *coelicolor* was identified in previous DNA affinity capturing assays byPark, Yang [23], binding to the promoter of red, the pathway specific regulator of the undecylprodigionines biosynthesis pathway. Rok7B7 has also been demonstrated to influence antibiotic production and xylose utilisation in *S*. *coelicolor* [24]. However, specific DNA-binding of Rok7B7 could not be detected in electrophoretic mobility shift assays (EMSA).

Recently we performed DNA-affinity capturing assays with the host strain *S*. *coelicolor* M512(nov-BG1) using promoter regions of the heterologously expressed novobiocin biosynthetic gene cluster from *Streptomyces niveus* [25, 26]. The promoter regions of the two regulatory genes of the *S*.*niveus* novobiocin biosynthetic gene cluster, *novE* (PnovE) and *novG* (PnovG) and of the biosynthetic genes *novH-novW* (PnovH), were used to capture DNA-binding proteins. Among other proteins, some ROK-family regulators were identified binding in high intensities to one or more of the tested promoter regions. RokB (Sco6115) was found to bind specifically and in high intensities to the promoter region of novH. This study describes the influence of RokB on heterologous novobiocin production, defines a DNA-binding motif and identifies putative members of the RokB regulon in *S*. *coelicolor*.

## Results

### Overexpression and deletion of *rokB* in the heterologous host reduce novobiocin production

The recently detected possible involvement of ROK-family regulator RokB (Sco6115) in the regulation of novobiocin biosynthesis [26] poses the question, if a homologous ROK protein exists in the native producer strain *S*. *niveus* NCIMB 11891. To answer this question, blast searches were performed for all 13 ROK-family proteins of *S*. *coelicolor* that contain a DNA binding domain against proteins from the native producer strain [27] (S1 Table). The result of this blast search demonstrates that some ROK regulators are highly conserved in both strains, as e.g. Rok7B7 (SCO6008) or SCO6566 with 87% and 89% aa identity, respectively, whereas others are not. RokB exhibits a low similarity to the *S*. *niveus* ROK protein M877_38045 (42% aa identity). This low similarity represents most likely not a functional homologue, as M877_38045 is also similar to a second ROK regulator from *S*. *coelicolor*, SCO7486, with 46% aa identities. As there is no clear homologue of RokB in *S*. *niveus*, it is intriguing to study its effects on novobiocin production in the heterologous host strain *S*. *coelicolor* M512.

To investigate the influence of RokB on novobiocin production in *S*. *coelicolor* M512 containing the *S*. *niveus* novobiocin biosynthetic gene cluster (*S*. *coelicolor* M512(nov-BG1)), *rokB* overexpressing as well as *rokB* deletion mutants were constructed.

**Fig 1:**
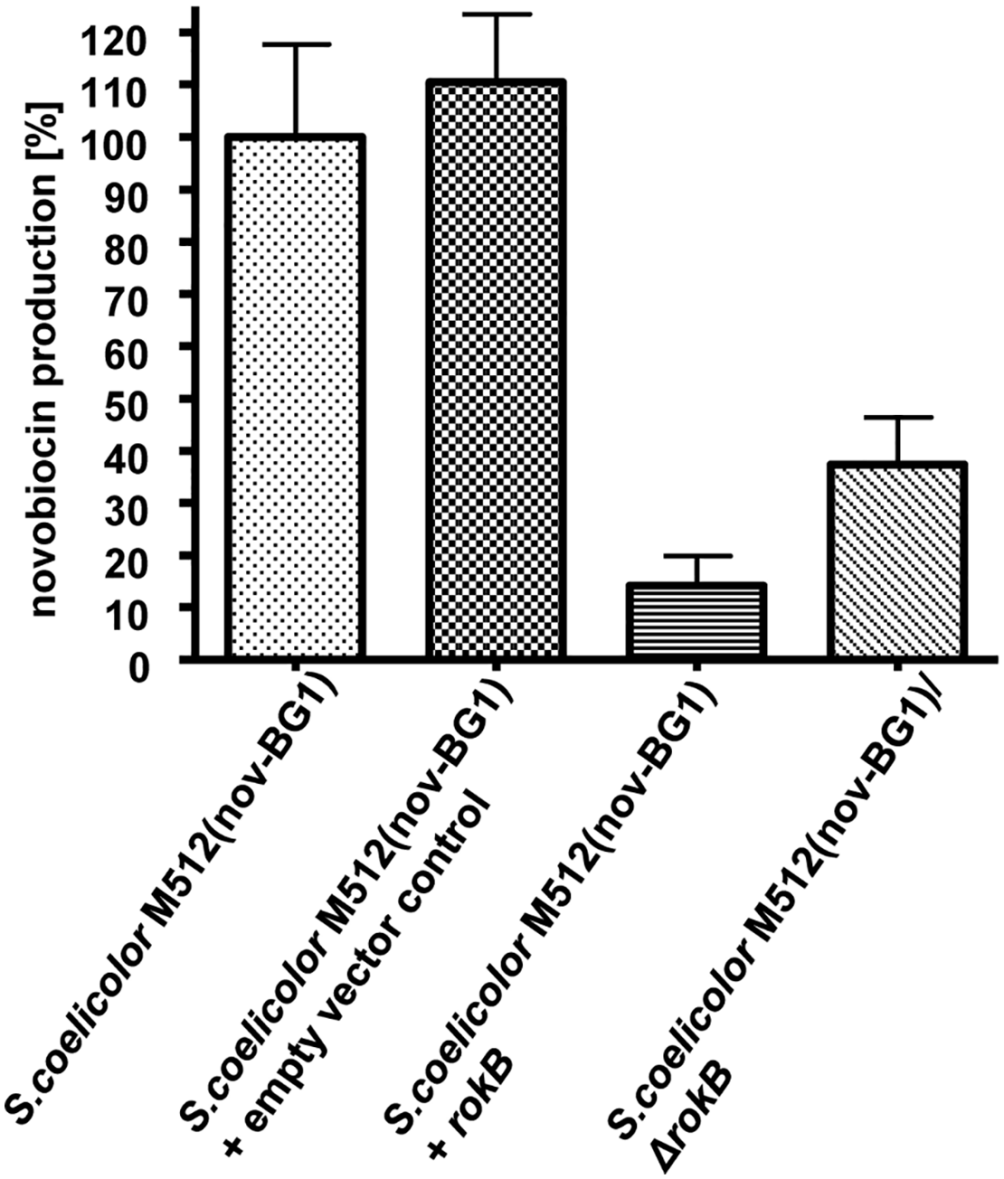
Novobiocin production of *rokB* deletion and overexpression mutants. Novobiocin production of *S*. *coelicolor* M512(nov-BG1)/pUWL-apra-oriT 1–6, *S*. *coelicolor* M512(nov-BG1)/pPBB12 1–5 (+*rokB*) and *S*. *coelicolor* M512(nov-BG1)/Δ*rokB* 1–3 compared relatively to *S*. *coelicolor* M512(nov-BG1) novobiocin production. Error bars indicate standard deviations between individual clones.

For overexpression, *rokB* was cloned into the self-replicating pUWL-apra-oriT vector under control of the constitutive strong promoter *ermE*p*. The resulting construct pPBB12 was introduced into *S*. *coelicolor* M512(nov-BG1) and five single clones were analysed for their novobiocin production and compared to *S*. *coelicolor* M512(nov-BG1) and *S*. *coelicolor* M512(nov-BG1) containing the empty pUWL-apra-oriT vector (Fig 1). Overexpression of *rokB* resulted in a drastic reduction of novobiocin production to 14% when compared to *S*. *coelicolor* M512(nov-BG1) and 13% compared to the empty vector control. This corresponds to the classic function of ROK regulatory proteins as transcriptional repressors.

To confirm this function, three independent deletion mutants of *rokB* were generated by Red/ET-mediated recombination in *S*. *coelicolor* M512. To avoid possible downstream effects of the knock-out cassette, the cassette containing the apramycin resistance gene was removed by FLP mediated recombination [28]. This created an in-frame deletion containing an 81 bp scar sequence. All three independent deletion mutants did not differ in growth and appearance from *S*. *coelicolor* M512 when cultivated in CDM medium. Subsequently, cosmid nov-BG1 containing the novobiocin biosynthetic gene cluster was introduced into each of the three deletion mutants. Two clones of each transformation were analysed for their novobiocin production and compared to *S*. *coelicolor* M512(novBG-1). Surprisingly *rokB* deletion reduced novobiocin production (Fig 1) to 37% compared to *S*. *coelicolor* M512(nov-BG1).

These results suggest that RokB might have both, a direct and an indirect regulatory effect on novobiocin production, but it remains unclear if RokB acts as an activator or repressor of the gene cluster. Identification of the binding site of RokB in the promoter region upstream of *novH* (PnovH) could help to solve this question.

### Identification of a RokB binding site in the promoter region of *novH*

To confirm RokB binding to the promoter region PnovH, *rokB* was cloned into the vector pGEX-6P-1 and the GST tagged protein (GST-RokB) was purified from *E*. *coli* Rosetta2^™^ (DE3)pLys. After cleavage of the GST-tag, RokB was tested in electrophoretic mobility shift assays (EMSA) for its DNA binding ability to PnovH. Despite trying several conditions, no specific binding to PnovH could be observed by EMSA (data not shown).

Binding of Sco6115 was originally observed in a DNA affinity capturing assay with protein extract from *S*. *coelicolor* M512 (nov-BG1). To verify the functionality of purified RokB, the experiment was repeated as described before [26], testing the promoter regions PnovE, PnovG, PnovH and PhrdB, but using purified RokB instead of protein extract from *S*. *coelicolor* M512 (nov-BG1). This experiment confirmed the specific binding of RokB to the 565 bp promoter region of the novobiocin biosynthetic genes PnovH (S1 Fig).

**Fig 2:**
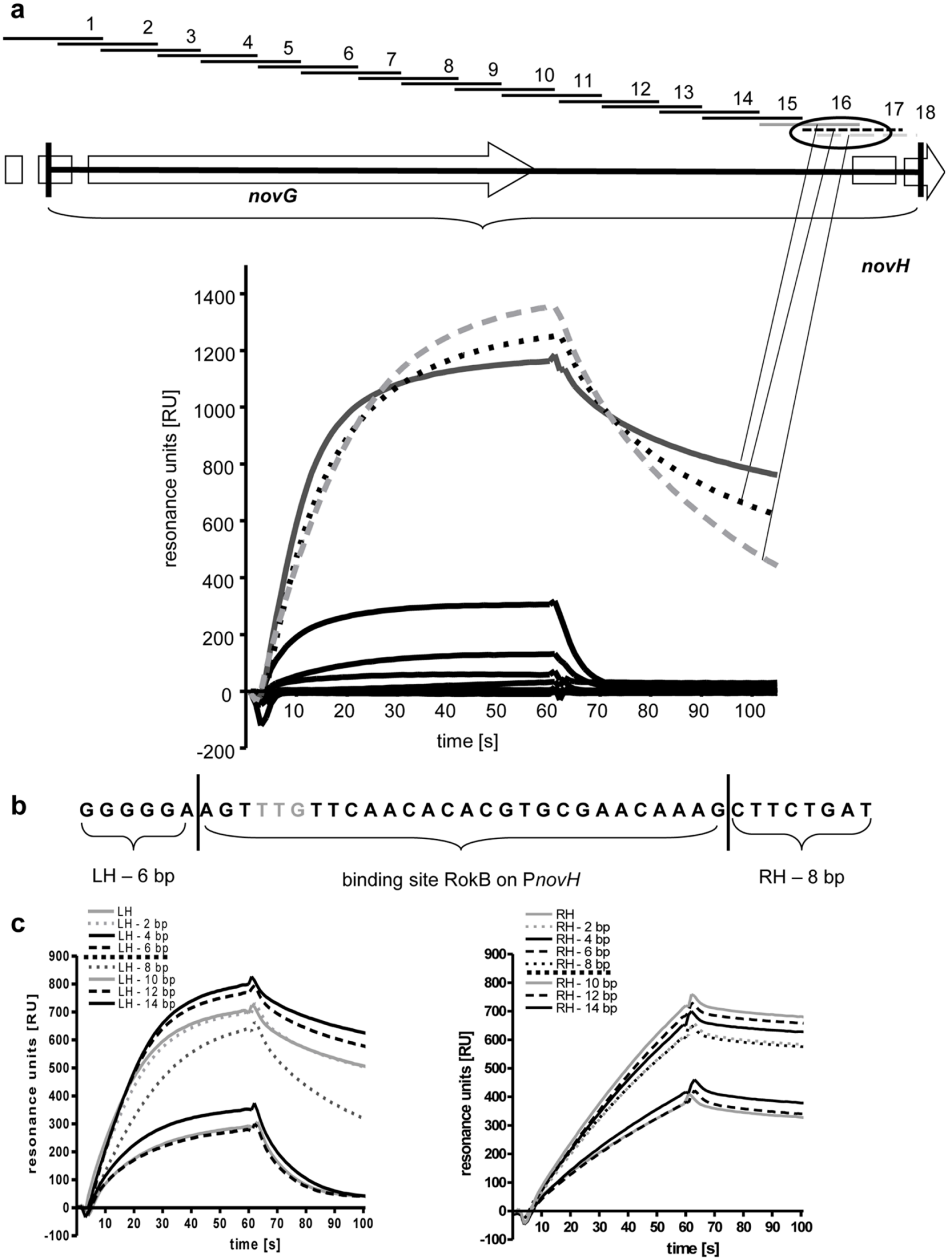
SPR experiments with RokB and PnovH. a Scheme of the promoter of *novH* with localisation of the tested DNA fragments (oligo 1–18) on PnovH and binding curves of RokB to the tested oligonucleotides. b Binding site of RokB on PnovH. Boundaries determined by SPR spectroscopy are indicated by vertical lines. Start codon of *novH* in grey (TTG). c SPR binding curves of RokB to 42 bp starting sequence shortened by 2 bp each for determination of the left-hand and right hand boundary of the RokB binding site on PnovH.

Compared to EMSA experiments, surface plasmon resonance (SPR) spectroscopy is a more sensitive method to study protein-DNA interactions. In contrast to EMSA, which requires gel electrophoretic separation of DNA fragments, SPR spectroscopy measures even transient protein-DNA complex formation in real-time in a micro-flow cell under stable buffer conditions. SPR spectroscopy was performed to identify the binding site of RokB within the promoter region PnovH with the recently described ReDCaT method [29], which enables subsequent testing of protein binding to different DNA fragments by using one single streptavidin covered sensor chip. The 565 bp sequence of PnovH was divided into 18 DNA fragments, each 60 bp long and overlapping by 30 bp using the program POOP [29], with fragment 18 being the end fragment, overlapping with fragment 16 and 17, due to the calculation of POOP A 60 bp DNA sequence from the promoter of the vegetative sigma factor *hrdB* (PhrdB-NC) served as negative control in the reference flow cell. Each of the 18 DNA fragments (oligo 1–18) was subsequently tested for RokB binding. Indeed, binding could be observed to three overlapping DNA fragments (oligos 16, 17 and 18, Fig 2a), whereas no considerable interaction of RokB was observed with the reference DNA, nor with the remaining 15 DNA fragments of PnovH. This indicated that the putative RokB binding site is located on the overlapping 42 bp region of fragments 16, 17 and 18.

Further characterization of the binding site followed by truncating this 42 bp region from the right hand boundary (RH) as well as from the left hand boundary (LH), to determine the exact binding site of RokB. Starting with the sequence 5′-GGGGG AAGTT TGTTC AACAC ACGTG CGAAC AAAGC TTCTG AT-3′ the binding site was determined by truncating first the left hand boundary repeatedly by 2 bp until LH– 14 bp. Interaction of RokB with each fragment was determined by SPR (Fig 2b and 2c). At DNA fragment LH– 8 bp association was similar to the DNA fragments LH until LH– 6 bp, but dissociation was noticeably faster, indicating that the binding site was not fully existent anymore. From DNA fragment LH– 10 bp on to LH– 14 bp, association was reduced by > 50% compared to LH until LH -8 bp and total dissociation was reached quickly. With these results, the left hand boundary for the RokB binding site on PnovH was set at LH– 6 bp. The same procedure was applied to the right hand boundary of the binding site. DNA fragments from RH until RH– 14 bp were tested for their RokB binding. DNA fragments RH until RH– 8 bp had similar binding curves, concerning association and dissociation, whereas for DNA fragments RH– 10 bp until RH– 14 bp DNA binding was visibly reduced. This indicates that the complete binding site is only present on DNA fragments RH until RH—8. The 28 bp binding site for RokB on PnovH was thus determined to be 5′-AGT**TTG**TTCAACACACGTGCGAACAAAG-3′ at a resolution of 2 bp (Fig 2c). The binding site contains an inverted repeat (underlined) and overlaps with the *novH* start codon (bold). We speculate that, by overlapping the *novH* start codon, RokB blocks transcription of all biosynthetic genes from *novH* to *novW*. This hypothesis would explain the observed reduction of novobiocin production after overexpressing RokB in the producer strain. Still the reduction of novobiocin production in the *rokB* deletion mutant needs to be explained. Studying the function of RokB in *S*. *coelicolor*, by *e*.*g*. the identification of RokB binding sites in the genome, might help to understand this phenomenon.

### Identification of RokB binding sites on the genome of *S*. *coelicolor*

**Fig 3:**
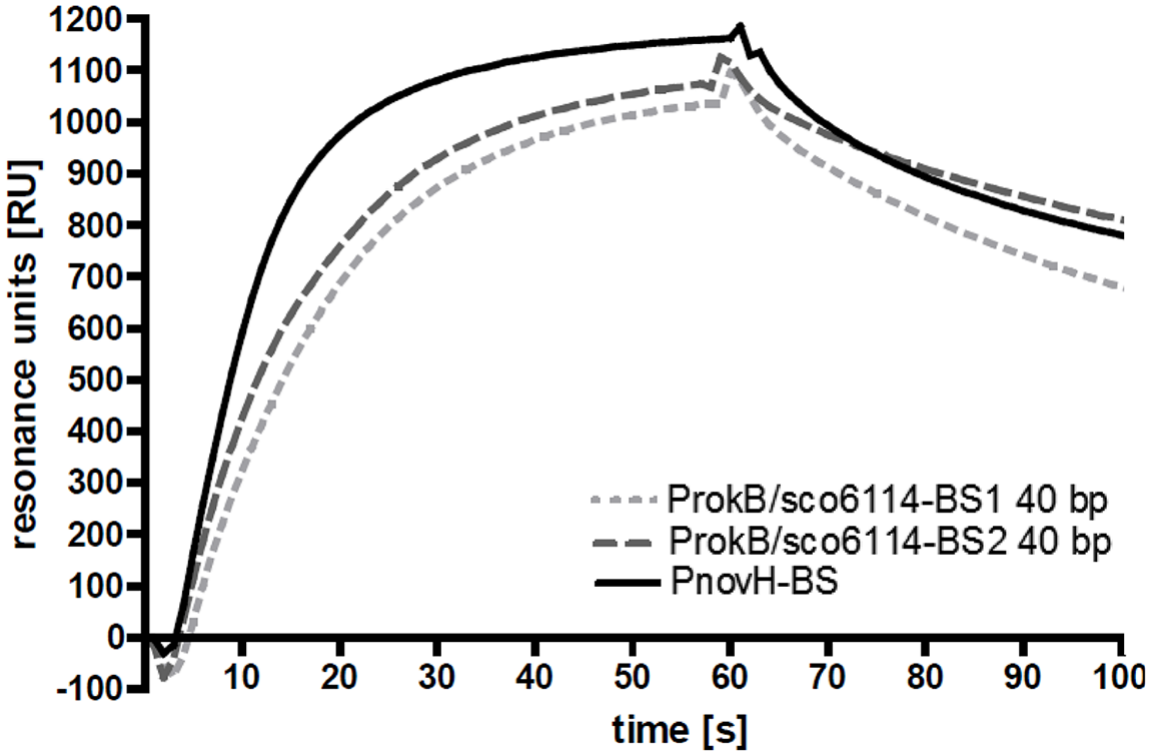
SPR binding curves with RokB and PnovH-BS, ProkB/sco6114-BS1 40 bp and ProkB/sco6114-BS2 40 bp.

Transcriptional regulators are often auto-regulating or regulating adjacent genes. Therefore the identified binding site was used to search a 13 kbp DNA region of the *S*. *coelicolor* genome enclosing the *rokB* promoter sequence for possible further binding sites by the RSA-tool *patser* [30]. Two possible binding sites were identified in the 210 bp intergenic, bidirectional promoter region between *sco6114* and *rokB*. The putative binding sites were named ProkB/sco6114-BS1 and ProkB/sco6114-BS2. 40 bp long DNA fragments containing the potential binding sites were tested for RokB binding by SPR as described above. RokB indeed binds both binding sites on its own bidirectional promoter region with similar association and dissociation curves as for its interaction with the binding site on PnovH (Fig 3).

**Fig 4:**
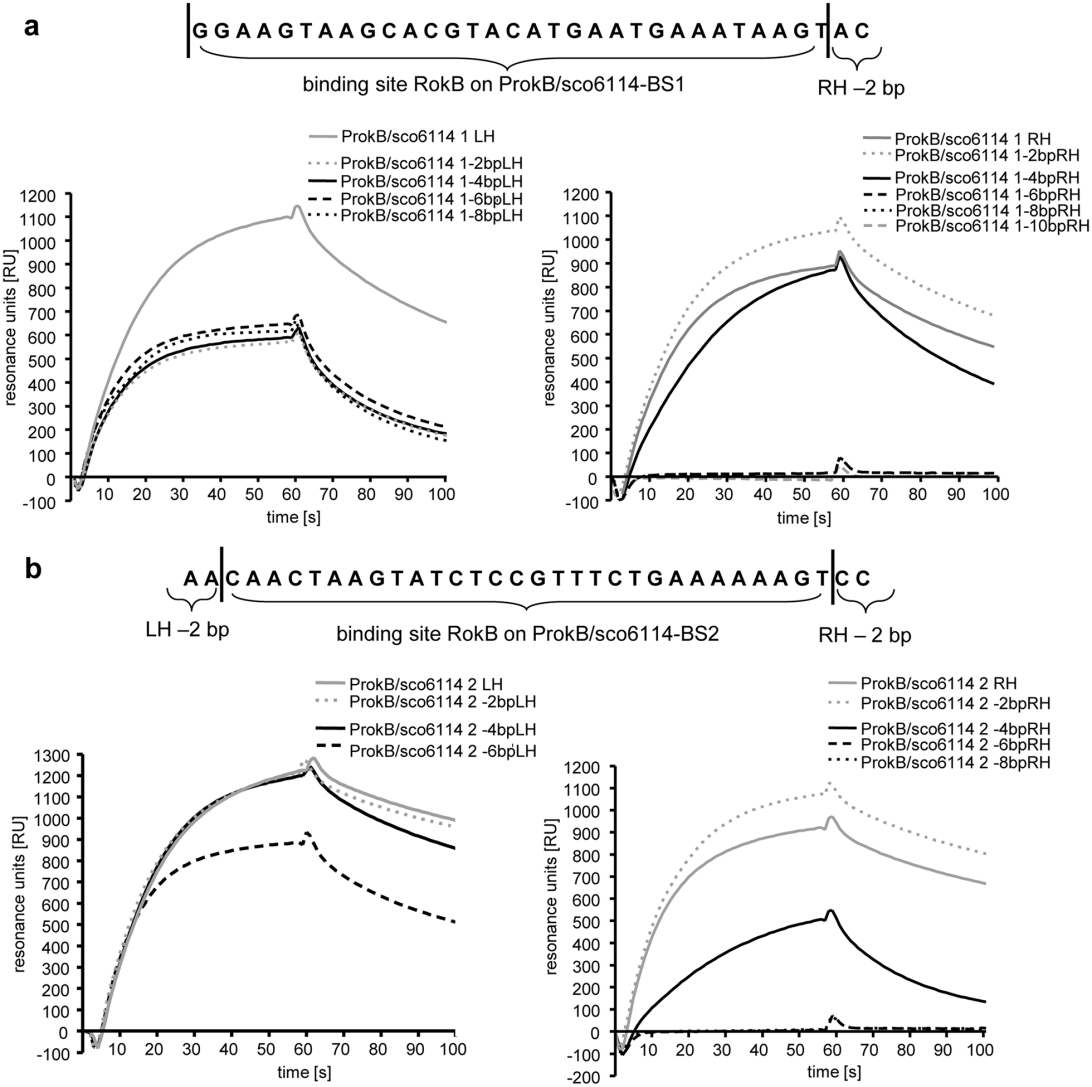
a Binding site of RokB on ProkB/sco6114-BS1. Boundaries are indicated by vertical lines. SPR binding curves of RokB to 33 bp starting sequence shortened by 2 bp each for determination of the left and right-hand boundary of the binding site. b Binding site of RokB on ProkB/sco6114-BS2. Boundaries are indicated by vertical lines. SPR binding curves of RokB to 34 bp starting sequence shortened by 2 bp each for determination of the left and right-hand boundary of the binding site.

Further characterization was accomplished by determining the right hand and left hand boundaries of the binding sites. The approach was according to the identification of the RokB binding site on PnovH. Two DNA fragments of 33 bp (5′-GGAAG TAAGC ACGTA CATGA ATGAA ATAAG TAC-3′) and 34 bp (5′-AACAA CTAAG TATCT CCGTT TCTGA AAAAA GTCC-3′) were used as starting sequences, truncated repeatedly by two bp and RokB interaction was measured by SPR (Fig 4). The following binding sites were identified for RokB on the bidirectional promoter region *sco6114/rokB* with a resolution of 2 bp: a 31 bp region 5′-GGAAG TAAGCACGTAC**AT****G**AATGAAATAAGT-3′ (ProkB/sco6114-BS1) starting at -16 bp of the *rokB* start codon (bold) and overlapping it, and a 30 bp region 5′-CAACTAAGTATCTCCGTTTCTGAAAAAAGT-3′ (ProkB/sco6114-BS2) located 136 bp to 105 bp upstream of *rokB* and at a distance of 104 bp to 75 bp from the *sco6114* start codon, respectively. Interestingly these two new binding sites for RokB on the genome of *S*. *coelicolor* contain no perfect inverted repeat, as it is the case for the binding site of RokB identified on the heterologously introduced novobiocin biosynthetic gene cluster. The binding sites are rather characterized by A-rich regions at the right and left hand of the binding site as well as a 5′-TVCVT-3′ sequence in its centre (underlined).

**Fig 5:**
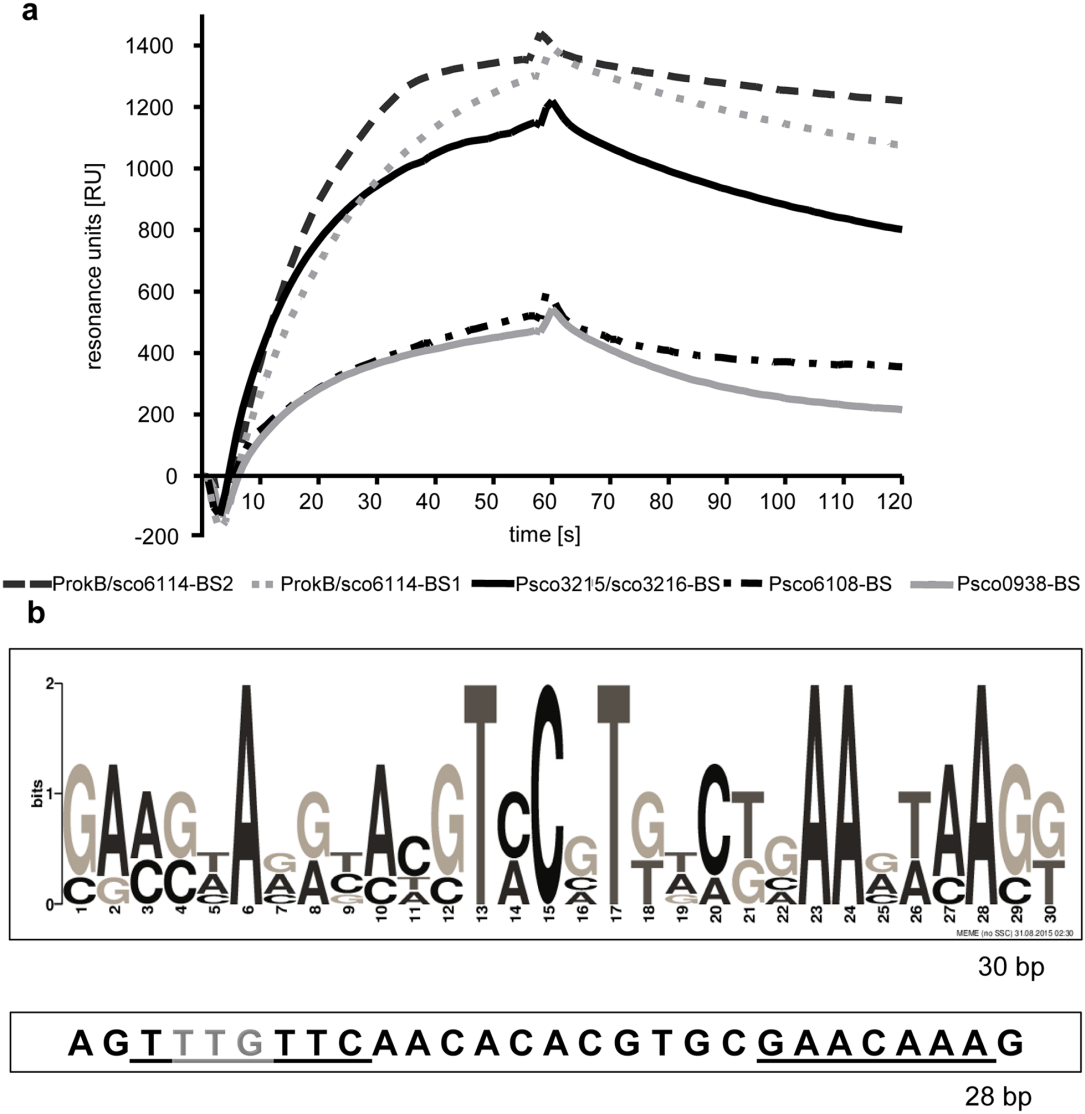
RokB binding sites on the genome of *S*. *coelicolor*. a SPR binding curves with RokB and ProkB/sco6114-BS1, ProkB/sco6114-BS2, Psco3215/3216-BS, Psco6108-BS, Psco0938-BS. b Binding motif of RokB on ProkB/sco6114-BS1, ProkB/sco6114-BS2, Psco3215/3216-BS, Psco6108-BS, Psco0938-BS, calculated by MEME and binding site of RokB on PnovH. Inverted repeat is underlined, TTG Startcodon of *novH* depicted in grey.

The knowledge of two genuine binding sites of RokB facilitated a wider search for further binding sites in the genome of *S*. *coelicolor* to identify possible members of the RokB regulon. The program PREDetector [31] was used to generate a matrix based on the two newly identified binding sites, which was then applied to a genome-wide search for further binding sites. The seven hits with the highest calculated similarity score (S2 Table) were tested for RokB binding by SPR spectroscopy. Five hits did not show any binding in SPR spectroscopy experiments. For two fragments upstream of *sco6108* (Psco6108-BS) and upstream of *sco0938* (Psco0938-BS) interactions with RokB were detectable (Fig 5), although weaker than for the previously identified binding sites. Psco6108-BS is a 30 bp sequence (5′-GAAGAAGGGAAGTACGTTGCTCAACAAAGG-3′) positioned at +18 bp from the start codon of *sco6108* and thus overlapping the coding sequence of this gene. The second binding site Psco0938-BS comprises 30 bp (5′-GACGCACACACGTCCGTGACGGAAGTCACG-3′) and is positioned at -78 bp from the start codon of *sco0938*. The four genomic binding sites were again used to generate a new matrix and to perform a genome wide search for further binding sites with the program PREDetector. Six hits with the highest calculated similarity scores (S2 Table) were tested for RokB binding, resulting in the identification of one more binding site, located in the bidirectional promoter region of *sco3215/sco3216* (Psco3215/3216-BS). Its interaction with RokB showed a similar binding curve as measured for ProkB/sco6114-BS1 and ProkB/sco6114-BS2 (Fig 5a). The binding sequence (5′-GGCCAAGATCCGTCCCTGTCGAAAGTAAGG-3′) is situated at -147 bp from the start codon of *sco3216* and at -176 bp from the start codon of *sco3215*.

The intensity of the interaction of RokB with its binding sites was tested by increasing the sodium chloride concentration in the binding buffer to 250 mM, instead of 100 mM sodium chloride, as used for all previous screening experiments. Solely the interactions between RokB and the binding sites ProkB/sco6114-BS1, ProkB/sco6114-BS2 and PnovH-BS withstanded the higher sodium chloride concentration (data not shown). Binding of RokB to Psco6108-BS, Psco0938-BS and Psco3215/3216-BS was not detectable at 250 mM sodium chloride. These findings suggest that the genomic binding sites ProkB/sco6114-BS1 and ProkB/sco6114-BS2 are better conserved than the other three identified binding sites. As ten of the predicted 13 sequences did not show binding of RokB, the search for further binding sites on the genome was not needed. A binding motif was calculated by MEME [32] (Fig 5b). It illustrates the conserved TVCVT motif in the centre of the binding site and the A-rich regions at the boundaries.

### A possible involvement of RokB in amino acid metabolism

**Fig 6:**
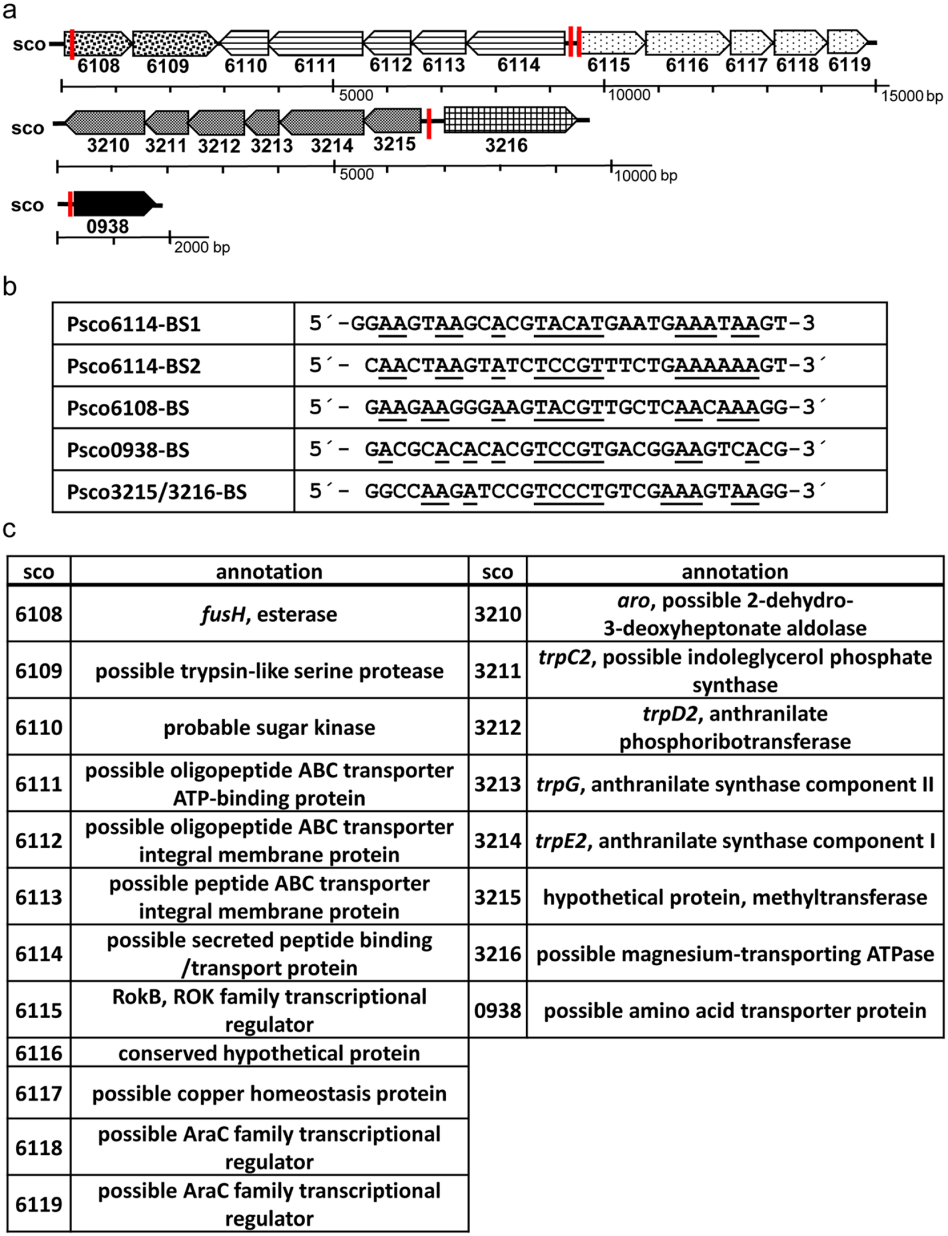
Binding sites of RokB in the Genome of *S*. *coelicolor*. a Location of RokB binding sites (marked by vertical bars) in the promoter regions of the respective operons. b Sequences of the 5 identified genomic RokB Binding sites. c Annotations of genes belonging to a possible RokB regulon.

Taking a closer look at the operons controlled by RokB, the most notable fact is that most genes seem not to belong to sugar metabolism, as it has mainly been reported for regulons controlled by ROK-family regulators. Instead the predicted functions of the genes suggest an involvement of RokB in amino acid metabolism. Fig 6 shows the gene clusters of the operons to which promoters RokB binds and depicts the localisation of the binding site.

The first two genes, *sco6108* and *sco6109*, form a possible operon in direct neighbourhood of *rokB*. *Sco6108* encodes for FusH, a highly specific esterase, which deacetylates and thereby inactivates the steroid antibiotic fusidic acid [33]. It was also found to belong to the group of ADP-ribosylated proteins [34]. Sco6109 shows homologies to secreted trypsin-like serine proteases and could be involved in extracellular protein degradation.

The next two operons are regulated from the bidirectional promoter Psco6114/rokB and include the regulator itself. The operon including the genes *rokB* (*sco6115*) to *sco6119* encodes besides RokB for two putative AraC family regulatory proteins, a putative copper homeostasis protein and a conserved hypothetical protein. While for these proteins an assignment to amino acid metabolism is not possible, the operon sco6114-sco6110 transcribed in opposite direction encodes a putative amino acid transporter. Sco6114 is annotated as possible secreted peptide binding / transport protein with similarity to e.g. oligopeptide-binding protein OppA from *Bacillus subtilis*. Sco6113 to Sco6111 belong to a probable oligopeptide ABC transporter and Sco6110 is annotated as probable sugar kinase with a Pfam match to entry PF00480 ROK, ROK family and match to Prosite entry PS01125 ROK family signature, without regulatory DNA-binding domain.

Sco0938 is annotated as an amino acid transporter and shares 46% amino acid identities to Sco6114. This type of amino acid transporter is highly conserved in different *Streptomyces* species.

The second bidirectional promoter region controls the transcription of *sco3216*, which has similarities to cation-transporting ATPases involved in Mg-transport, and the operon spanning from *sco3215* to *sco3210*. The latter genes are organized in one operon with maximum 3 bp intergenic region between each gene and are predicted to be involved in tryptophan synthesis. Sco3210 has similarities to a 2-dehydro-3-deoxyheptonate aldolase, which catalyses the first step of the shikimate pathway and thereby supplies precursors for phenylalanine, tyrosine and tryptophan biosynthesis from the primary metabolism. Sco3214 and Sco3213 share homologies with the proteins TrpE2 and TrpG, the anthranilate synthase components I and II. These enzymes catalyse the first step of tryptophan synthesis, the synthesis of anthranilate from chorismate. Sco3212 is annotated as TrpD2, a probable anthranilate phosphoribotransferase catalysing the next step to phosphoribosyl anthranilate, and Sco3211 is annotated as TrpC2, a probable indoleglycerol phosphate synthase and could form indole-3-glycerol phosphate, a direct precursor of tryptophan. Sco3215 shares homologies with SAM dependent methyltransferases.

Especially the latter genes involvement in the shikimate pathway pose a possible conflict with novobiocin biosynthesis. The shikimate pathway is involved in the biosynthesis of phenylalanine, tyrosine and tryptophan, with L-tyrosine being the main precursor for novobiocin biosynthesis. It was postulated that the deletion of *rokB* might lead to an impairment of amino acid supply and of the shikimate pathway and by this leads to the reduced novobiocin production observed for the deletion mutants. To examine this hypothesis the *rokB* deletion mutants were cultivated together with the *rokB* overexpressing mutants and the unmodified heterologous producer strain in feeding experiments with casamino acids and L-tyrosine.

**Fig 7:**
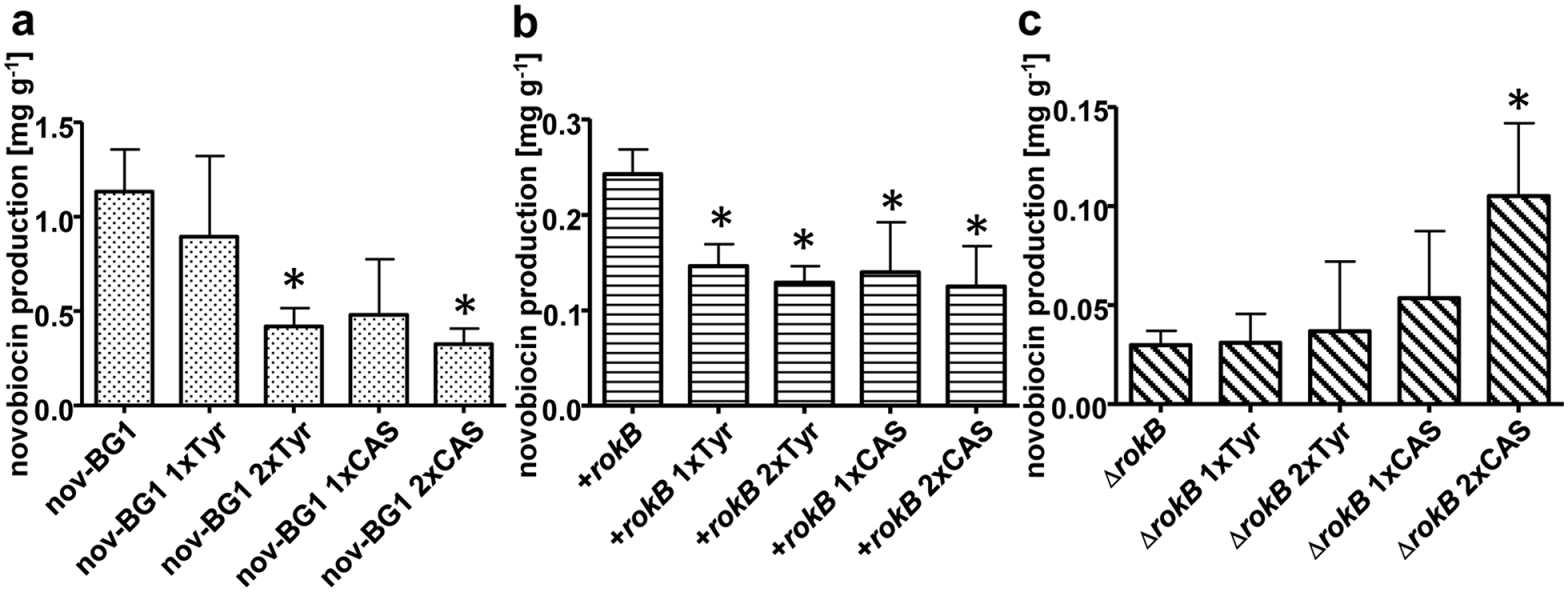
Novobiocin production after feeding with L-tyrosine and casamino acids. a Novobiocin production [mg g^-1^ dry weight] of *S*. *coelicolor* M512(nov-BG1) unfed, after feeding of 1mM L-tyrosine once and twice and after feeding of 1g L-1 casamino acids once and twice. b *S*. *coelicolor* M512(nov-BG1)/ pPBB12 (+*rokB*) 1–5 unfed, after feeding of 1mM L-tyrosine once and twice and after feeding of 1g L-1 casamino acids once and twice. c *S*. *coelicolor* M512(nov-BG1) Δ*rokB* 1–3 unfed, after feeding of 1mM L-tyrosine once and twice and after feeding of 1g L-1 casamino acids once and twice. Error bars indicate standard deviation between triplicated measurements.

Feeding of L-tyrosine and casamino acids to the *rokB* overexpressing mutants and the wild type strain significantly reduced novobiocin biosynthesis. In contrast, in the *rokB* deletion mutants, novobiocin production was slightly increased by L-tyrosine and the effect of casamino acids was even more pronounced. Feeding of 1 g L^-1^ casamino acids either once at the beginning of cultivation or twice at the beginning and after 36 h of cultivation to the production medium increased novobiocin production in the *rokB* deletion mutants to 170% and 369%, respectively. In contrast, at the same time there was a reduction to 58% and 51% in the *rokB* overexpressing mutants and to 42% or 33% in *S*. *coelicolor* M512(nov-BG1) compared to the unfed strains, respectively (Fig 7). These results suggest that RokB indeed is regulating amino acid transport and metabolism and that the observed reduction of novobiocin production in the *rokB* deletion mutants is a result of this. External feeding of tyrosine and small peptides (casamino acids) can partly restore the precursor supply for novobiocin biosynthesis in the *rokB* deletion mutants.

## Discussion

In this study we have shown that a ROK-family regulator, RokB, from *S*. *coelicolor* negatively regulates the production of novobiocin from a heterologously introduced novobiocin biosynthetic gene cluster. We could prove that RokB binds to a 28 bp binding site PnovH-BS, overlapping with the start codon of the first biosynthetic gene *novH*, and we suggest a mechanisms by which *rokB* overexpression and deletion may influence novobiocin production. Further studies, including transcription analysis by RT-qPCR, will be required to confirm these hypotheses.

Further five RokB binding sites were identified on the genome of *S*. *coelicolor*, thus defining 22 genes that belong to a putative RokB regulon. The regulon was suggested to be involved in amino acid metabolism and transport, thus impairing those processes in the deletion mutant and resulting in reduced novobiocin production. Feeding experiments with tyrosine and casamino acids could strengthen this hypothesis.

Our attraction originally was driven on RokB, as we identified it in a DNA affinity capturing assay [26]. It was one of the few proteins binding with high specificity and intensity to the promotor region of the biosynthetic genes *novH*-*novW* (PnovH) on the heterologously introduced novobiocin biosynthetic gene cluster from *S*. *niveus* NCIMB11891. Intriguingly, neither does *S*. *coelicolor* contain a native aminocoumarin biosynthetic gene cluster, nor could a close RokB homologue be found in *S*. *niveus*. Another ROK-family protein, Rok7B7 from *S*. *coelicolor*, has been identified before in a DNA affinity capturing assay, binding upstream of the pathway specific regulatory gene *redD* in the prodiginine biosynthetic gene cluster [23]. Its involvement in antibiotic production, xylose utilization and carbon catabolite repression was demonstrated by Swiatek, Gubbens (24), but no direct binding to the promoter regions were detected. We therefore studied the influence of *rokB* on novobiocin production by overexpression and deletion in the heterologous producer strain. Surprisingly, both overexpression and deletion of *rokB* resulted in a significant reduction of novobiocin production in *S*. *coelicolor* M512(nov-BG1).

A RokB binding site (PnovH-BS) in the promoter region regulating the transcription of *novH*-*novW* could be identified and characterized by SPR experiments. It is formed by an inverted repeat with the sequence 5′-TTTGTTC-11X-GAACAAA-3′ and overlaps with the start codon of *novH*. The reduction of novobiocin production in the overexpression mutants may be explained by binding of RokB to PnovH-BS, thereby blocking the start codon of *novH* and consequently preventing transcription of *novH* and all following novobiocin biosynthetic genes. The organisation of PnovH-BS is similar to previously reported binding sites of ROK-family regulators, as e.g. for RokA from *S*. *pneumoniae* D39, which has a T-rich region on the 5´-end of the binding site, and an A-rich region at the 3´-end [11]. The binding site of CysR in *C*. *glutamicum* ATCC 13032 has a similar organisation [12], as well as the binding sites of Mlc and NagC from *E*. *coli* [35], the binding site of RafR from *B*. *breve* UCC2003 [15], or all described binding sites from the phylum *Thermotogae* [18].

Further five binding sites of RokB could be identified on the genome of *S*. *coelicolor*. The characterization of these binding sites by SPR spectroscopy defined a slightly different binding motif than identified with PnovH-BS. Although binding curves and maximal resonance units are similar for the two binding sites of RokB on its own bidirectional promoter compared to those for PnovH-BS, they differ in length (+2 bp for ProkB/sco6114-BS1, +3 bp for ProkB/sco6114-BS2) and organisation. The same organisation could be confirmed for the three further binding sites Psco0938-BS, Psco6008-BS and Psco3215/3216-BS. These five binding sites represent a new unusual binding motif for ROK-family regulators, with a conserved TVCVT motif in the centre of the binding site and A-rich regions at the boundaries (Fig 6b). Further investigation including crystal structures of RokB, bound to its DNA targets, could solve the question, how exactly RokB can bind to those differently organised binding sites. The binding sites identified in this study are the first reported DNA-binding sites for a ROK-family regulator from *S*. *coelicolor*.

By analysing the genes putatively regulated by RokB in *S*. *coelicolor*, there is evidence to suggest that RokB is involved in the regulation of amino acid metabolism and transport. Thereby it might affect precursor supply for secondary metabolism, including novobiocin biosynthesis, as L-tyrosine is an essential precursor for novobiocin biosynthesis [36]. Feeding of L-tyrosine and casamino acids had a positive effect on novobiocin production in the *rokB* deletion mutants, while decreasing its formation in the *rokB* overexpressing mutants and in the heterologous producer strain *S*. *coelicolor* M512(nov-BG1). Zhao and colleagues demonstrated that feeding of tryptophan, tyrosine and phenylalanine reduced production of rapamycin in *Streptomyces hygroscopicus* [37]. The authors discuss a possible feedback inhibition of those amino acids on the biosynthesis of shikimic acid from phosphoenolpyruvate and erythrose-4-phosphate to 3-deoxy-d-arabino-heptulosonate-7-phosphate (DAHP) as reason for this effect. The first step of biosynthesis of shikimic acid, the synthesis of DAHP, is tightly regulated, in contrast to the other biosynthetic proteins, which often are expressed constitutively, as described by Kramer, Bongaerts [38]. This might explain the decrease of novobiocin production in the *rokB* overexpressing mutants, as well as in the wild type strain, when fed with tyrosine or casamino acids. As a putative member of the RokB regulon Sco3210 is one of two DAHP synthetases found in *S*. *coelicolor*. Sco3215 to Sco3211 are probably involved in tryptophan synthesis. Further putative RokB regulated genes are *sco6114* –*sco6110*, encoding for a possible oligopeptide transporter, *sco0938* encoding a possible peptide/amino acid transporter, and *sco6109* encoding a putative trypsin-like serine protease. Involvement of RokB in the regulation of those genes could explain the decrease of novobiocin production in the deletion mutants due to impaired amino acid precursor supply. Furthermore RokB would be involved in amino acid metabolism and thus different from most investigated ROK-family regulators which are involved in carbon catabolite repression and sugar metabolism. A possible involvement of ROK regulators in amino acid metabolism was reported previously for Rok7B7 in *S*. *coelicolor*, whose deletion resulted in upregulation of the branched chain amino acids leucine, isoleucine, and valine [24].

## Materials and Methods

### Bacterial strains, plasmids, cosmids and culture conditions

The *E*. *coli* and *Streptomyces* strains, plasmids, cosmids and DNA oligos used in this study are listed in S3 Table. *E*. *coli* strains were cultivated in liquid or on solid LB medium at 37°C. Media components were purchased from Carl Roth, Karlsruhe, Germany. Standard methods for cultivation, DNA isolation and manipulation were performed as described by Sambrook and Russel [39] and Kieser, Bibb [40]. DNA oligos were purchased from MWG Biotech AG, Eurofins Genomics, München, Germany.

### Deletion of *rokB* (*sco6115*)

Inactivation of *rokB* was performed using the PCR targeting system [28]. In a first step the *aac(3)IV* cassette of pIJ773 was amplified by PCR using the primer pairs RokBKO_F/RokBKO_R and used to replace *rokB* on cosmid 6F04 (SuperCos1-based, kanamycin-resistant, Kan^R^), which was kindly provided by Prof. Paul Dyson (Swansea university). Deletion of the *aac(3)IV* cassette from the resulting cosmids was carried out by using FLP-recombinase leaving an 81 bp scar region without start and stop codons. The resulting construct 6F04Δ*rokB* was introduced into *S*. *coelicolor* M512 by PEG mediated protoplast transformation [40]. Three individual Kan^R^ single crossover clones were further streaked out in order to obtain three independent Kan^S^ double crossover clones. Deletion of the genes was confirmed by colony PCR using primer pairs RokB_FTEST/RokB_RTEST and rokBGenF/ rokBGenR for verification of the deletion and sequencing to verify the in-frame deletion. Cosmid nov-BG1 was introduced into each double crossover mutant by triparental mating [40], resulting in *S*. *coelicolor* M512 Δ*rokB*(nov-BG1) 1–3. Introduction of nov-BG1 to each mutant was verified by colony PCR. For each of the three independent mutants two clones were analysed for novobiocin production.

### Overexpression plasmid construction

Using the primer pair RokBpUWLforw/RokBpUWLrev *rokB* was amplified from cosmid 6F04, introducing *Hin*dIII/*Spe*I restriction sites to the amplified gene. The PCR product was cloned into pGEM^®^-T cloning vector (Promega, Mannheim, Germany) and the sequence of the resulting plasmid confirmed by sequencing. Subsequently *rokB* was cloned into the vector pUWL-apra-oriT under control of the constitutive promoter *ermE*p*, taking advantage of the *Hin*dIII and *Spe*I restriction sites, and again confirmed by sequencing. The resulting plasmid pPBB12 and pUWL-apra-oriT (negative control) were conjugated into *S*. *coelicolor* M512(novBG-1) [40]. Exconjugants were verified by colony PCR using primer pairs pUWLtestF/pUWLtestR. Five and six single Apra^R^ clones (*S*. *coelicolor* M512(novBG-1)/pPBB12 1–5 and *S*. *coelicolor* M512(nov-BG1)/pUWL-apra-oriT 1–6) were analysed for their novobiocin production, respectivly.

### Analysis of novobiocin production

To improve reproducibility of novobiocin production, cultivation of all strains was performed in 24-square deepwell plates as described by Siebenberg et al. [41]. Strains were cultivated in CDM medium [42] using frozen homogenised mycelium as inoculum and harvested after nine days. For comparison of the deletion mutants, the novobiocin production of three independent mutants was measured in duplicates three times each with *S*. *coelicolor* M512(nov-BG1) as a control. For comparison of the overproducing mutants, novobiocin production of five independent mutants was measured three times each with *S*. *coelicolor* M512(nov-BG1)/pUWL-apra-oriT and *S*. *coelicolor* M512(nov-BG1) as a control. Analysis of novobiocin production by HPLC was carried out as described previously [43].

### Expression and purification of RokB

*RokB* was amplified from cosmid 6F04 using primers RokBfpGEX/RokBrpGEX introducing *Bam*HI/*Eco*RI restriction sites to the PCR product. The PCR product was cloned into pGEM^®^-T cloning vector (Promega, Mannheim, Germany). The resulting plasmid pPBB3 was verified by sequencing. The gene was subsequently cloned into the expression vector pGEX-6P-1 (GE Healthcare, Freiburg, Germany), taking advantage of the *Bam*HI and *Eco*RI restriction sites, and the resulting plasmid pPBB16 was verified by sequencing. *E*. *coli* Rosetta2^™^ (DE3)pLys (Novagen, Darmstadt, Germany) containing pPBB16 was cultivated in 2 L of TB broth (components purchased from Carl Roth, Karlsruhe, Germany) supplemented with 50 μg mL^-1^ carbenicillin and 50 μg mL^-1^ chloramphenicol at 37°C. At an OD_600_ of 0.7 temperature was adjusted to 20°C and isopropyl thiogalactoside was added to 0.5 mM final concentration. After additional 14 h of cultivation at 20°C the cells were harvested. The pellet was suspended in 2.5 mL g^-1^ cells of buffer A (50 mM Tris-HCl, pH 8, 500 mM NaCl, 10% glycerol, 1% Tween 20, 10 mM β-mercaptoethanol) supplemented with 0.5 mg mL^-1^ lysozyme and 0.5 mM phenylmethylsulfonyl fluoride. Cells were disrupted by sonication (Branson, Danbury, CT) at 4°C. The lysate were centrifuged (55000 × *g*, 45 min) and the supernatant was applied to affinity chromatography using an Äktapurifier^™^ platform (GE Healthcare, Freiburg, Germany) equipped with a GSTrap^™^ (34 μm, 1.6 × 2.5 cm) HP column (GE Healthcare, Freiburg, Germany). Buffer B (50 mM Tris-HCl, pH 8, 150 mM NaCl, 10% glycerol, 5 mM DTT) was applied as running buffer. The GST-tagged protein was subsequently eluted from the column with 10 mM glutathione in buffer B and collected by a Frac-920^™^ system (GE Healthcare, Freiburg, Germany). Glutathione was removed using a PD-10 column (GE Healthcare, Freiburg, Germany) according to the manufacturer's protocol. For cleavage of the GST-tag, the protein was incubated over night with 1 unit HRV3C protease per 100 μg protein (MoBiTec, Göttingen, Germany) at 4°C. The protein was purified from the cleaved GST-tag and protease using the Äktapurifier^™^ platform (GE Healthcare, Freiburg, Germany) equipped with a GSTrap^™^ (34 μm, 1.6 × 2.5 cm) HP column (GE Healthcare, Freiburg, Germany) and buffer B (50 mM tris-HCl, pH 8, 150 mM NaCl, 10% glycerol, 5 mM DTT). The GST-tagless protein was eluted immediately, while protease and the cleaved GST-tag remained on the column. A yield of 26.95 mg of purified RokB was obtained per litre of culture. RokB was stored at -80°C in aliquots.

### DNA affinity capturing assay

Promoter regions were amplified from genomic DNA of *S*. *coelicolor* M512(novBG-1) using primer pairs PnovEforward/PnovEreverse, PnovGforward/PnovGreverse, PnovHforward/PnovHreverse or PhrdBEforward/PhrdBreverse, respectively. Each reverse primer contained an additional linker sequence for subsequent biotinylation of the PCR products. The resulting DNA fragments PnovE (609 bp), PnovG (576 bp), PnovH (565 bp) and PhrdB (582 bp) were purified by gel electrophoresis and sequences were confirmed by sequencing. For biotinylation the fragments were amplified by PCR with the biotinylated primer DAC Biotin and the corresponding forward primer. For each experiment 122 μg of purified biotinylated promoter fragment was bound to 5 mg Dynabeads^®^ M-280 Streptavidin (Invitrogen, Darmstadt, Germany) according to the manufacturer's instructions. Beads were stored in TGED buffer (20 mM Tris HCl, 1mM EDTA, 100 mM NaCl, 0.01% Triton-X-100, 10% glycerol, 1 mM DTT, pH 7.5) at 4°C.

For each DNA affinity capturing assay, 100 μg mL^-1^ salmon sperm DNA (D1626 by Sigma Aldrich, Germany) was added to 58.8 μg purified RokB protein and incubated for 15 min on ice. 5 mg Dynabeads^®^ M-280 Streptavidin with coupled promoter region were added and gently shaken for 45 min at room temperature. Dynabeads^®^ M-280 Streptavidin were washed once with 500 μL TGED buffer, once with 500 μL TGED buffer containing 400 μg salmon sperm DNA to additionally remove unspecifically bound protein and once again with 500 μL TGED buffer. DNA binding protein was then eluted twice with 350 μL TGED buffer containing 2 M NaCl. Eluted protein from each tested promoter region was precipitated with 0.25 volumes of trichlorocetic acid and solved in 30 μL TGED buffer before analysis. All eluates were tested for presence of RokB by SDS Page.

### SPR spectroscopy

SPR spectroscopy was performed on a Biacore X System (GE Healthcare, Freiburg, Germany) at 20°C. BIA evaluation software (GE Healthcare, Freiburg, Germany) was used for data analysis. The recently developed ReDCaT method was applied [29]. It allows usage of one single Sensor Chip SA (GE Healthcare, Freiburg, Germany) for testing of different DNA fragments. The ´screening´ protocol was followed, with some slight variations [29]. Flow cell 1 was used as reference flow cell, containing an unspecific sequence from the *hrdB* promoter (PhrdB-NC), while flow cell 2 was the test flow cell. All samples were prepared, and all experiments were performed at a flow rate of 10 μL min^-1^ with an injection volume of 10 μL, in DNA binding buffer (100 mM NaCl, 0.2 mM EDTA, 0.005% (v/v) NP40, 10 mM HEPES (pH 7.4). Regeneration was performed with regeneration buffer (1 M NaCl, 50 mM NaOH).

All tested sequences are listed in the supplementary data (S3 Table). Each DNA fragment was tested twice. 1 μM test DNA and 1 μM RokB protein were used for injection at a flow rate of 10 μL min^-1^. Injection lasted 60 s, followed by 120 s washing with DNA binding buffer.

### Feeding of amino acids

For feeding of casamino acids the strains *S*. *coelicolor* M512 Δ*rokB*(nov-BG1) 1–3, *S*. *coelicolor* M512(novBG-1)/pPBB12 1–5 and *S*. *coelicolor* M512(novBG-1) were supplemented with 1 g L^-1^ Bacto^™^ casamino acids (Becton, Dickinson and Company, Heidelberg, Germany) either once, with one feeding at time point 0 h or twice with the first feeding at 0 h and a second feeding after 36 h growth. For feeding of L-tyrosine the strains *S*. *coelicolor* M512 Δ*rokB*(nov-BG1) 1–3, *S*. *coelicolor* M512(novBG-1)/pPBB12 1–5 and *S*. *coelicolor* M512(novBG-1) were supplemented with 1mM L-tyrosine either once, with one feeding at time point 0 h or twice with the first feeding at 0 h and a second feeding after 36 h growth. Casamino acids were solved in water, L-tyrosine was solved in 0.1 N HCl and each solution was filtered sterile through 0.22 μm PVDF membrane filters (Carl Roth, Karlsruhe, Germany). After addition of L-tyrosine the pH of the medium was checked to exclude effects of possible pH variation. Novobiocin production rates of the supplemented strains were compared to those of the unsupplemented cultures. Each cultivation was repeated three times.

## Supporting Information

S1 Fig DNA affinity capturing with purified RokB protein. SDS Page of eluted protein from PnovE, PnovG, PnovH and PhrdB.
(EPS)

S1 Table Putative ROK-family regulatory proteins found in the heterologous host strain *S*. *coelicolor* and their homologues from the original novobiocin producer strain *S*. *niveus*. (PDF)

S2 Table Binding sites suggested by PREDetector. a Binding sites suggested by PREDetector search with ProkB/sco6114-BS1 and ProkB/sco6114-BS2 as templates for matrix generation. These suggested binding sites were tested for interaction with RokB. Positive hits shadowed in grey and printed in bold type. b Binding sites suggested by PREDetector search, with ProkB/sco6114-BS1, ProkB/sco6114-BS2, Psco6108-BS and Psco0938-BS as templates for matrix generation. These suggested binding sites were tested for interaction with RokB. Positive hits shadowed in grey and printed in bold type.
(EPS)

S3 Table *E*. *coli* and *Streptomyces* strains, plasmids, cosmids and DNA oligonucleotides used in this study. (PDF)
