# Supplementary material for: Characterization of DNA Binding Sites of RokB, a ROK-Family Regulator from Streptomyces coelicolor Reveals the RokB Regulon
Source: PLoS One. 2016 May 4;11(5):e0153249. doi: 10.1371/journal.pone.0153249 (PMC4856308; doi:10.1371/journal.pone.0153249)
Supplement: S1 Table — (PDF) [file pone.0153249.s002.pdf]

Table S1: Putative ROK-family regulatory proteins found in the heterologous host strain *Streptomyces coelicolor* and their homologues from the original novobiocin producer strain *Streptomyces niveus*.

S. coelicolor	***S. niveus***	**Aa identity**
SCO0794	M877_03895	65 %
SCO1039	M877_37210	84 %
SCO1060	M877_05195	73 %
SCO1171	M877_05195	46 %
SCO1261	M877_35785	82 %
SCO2657	M877_27795	77 %
SCO2846	M877_26735	68 %
SCO6008(Rok7b7)	M877_08185	87 %
SCO6115(RokB)	M877_38045	42 %
SCO6566	M877_05555	89 %
SCO6600	M877_03895	60 %
SCO7486	M877_38045	46 %
SCO7543	M877_08185	33 %
