# Supplementary material for: Characterization of DNA Binding Sites of RokB, a ROK-Family Regulator from Streptomyces coelicolor Reveals the RokB Regulon
Source: PLoS One. 2016 May 4;11(5):e0153249. doi: 10.1371/journal.pone.0153249 (PMC4856308; doi:10.1371/journal.pone.0153249)
Supplement: S3 Table — (PDF) [file pone.0153249.s004.pdf]

Table S3: *E. coli* and *Streptomyces* strains, plasmids, cosmids and DNA oligonucleotides used in this study

a Bacterial strains, cosmids and plasmids used in this study

strain	relevant characteristics	source/reference
E. coli XL1Blue MRF`	*rec*A1 *end*A1 gyrA96 *hsd*R17 *sup*E44 *thi*-1 *rel*A1 *lac* [F´proAB *lacI*^{q}ZΔM15 Tn10, (Tet^{R})]	Stratagene, USA
E. coli ET12567	Strain triply defective in DNA methylation, Tet^{R}, Cml^{R} (*dam*$^{-}$, *dcm*$^{-}$, *nsd*M^{-})	(1)
E. coli Rosetta2 (DE3)pLysS	Host for the heterologous expression of proteins (F- *ompT hsdSB(rB-mB-) gal dcm* λ(DE3 [*lacI lacUV5-T7* gene *1ind1 sam7 nin5*]) pLysSRARE); Cm^{R}	Invitrogen, Darmstadt, Germany
E. coli BW25113	D(*araD-araB*)567 D*lacZ*4787::*rrnB-4;lacIP*-4000(*lacQ*) l-*rpoS369*(Am) *rph-1* D(*rhaDrhaB*)568 *hsdR514*	(2)
S. coelicolor M512	SCP1^{-}, SCP2^{-}, Δ*actII*-ORF4, Δ*redD*	(3)
S. coelicolor M512 (nov-BG1)	*S. coelicolor* M512 containing the novobiocin biosynthetic gene cluster, Kan^{R}	(4)
S. niveus NCIMB 11891	novobiocin producer, wild type	(5)
S. coelicolor M512 (nov-BG1) Δ*rokB (1), (2) and (3)*	*S. coelicolor* M512 containing the novobiocin biosynthetic gene cluster and an in-frame deletion of *rokB*, Kan^{R}, three independent mutants	this study
S. coelicolor M512 (nov-BG1)/ pUWL-apra-oriT (1) - (6)	*S. coelicolor* M512 containing the novobiocin biosynthetic gene cluster and the empty pUWL-apra-oriT plasmid, Kan^{R}, Apra^{R}, six independent exconjugants	this study
S. coelicolor M512 (nov-BG1)/ pPBB12 (1) - (5)	*S. coelicolor* M512 containing the novobiocin biosynthetic gene cluster and the *rokB* overexpressing plasmid, Kan^{R}, Apra^{R}, six independent exconjugants	this study
cosmids	description	source/reference
nov-BG1	SuperCos 1 based cosmid harbouring the novobiocin biosynthetic gene cluster, *oriT*, *tet*, *attP*, int ϴC31, Kan^{R}	(4)
6F04	SuperCos1 based Cosmid, *S. coelicolor* gene bank	(6)
6F04-Δ*rokB*-apra	6F04, *rokB* replaced through FRT-*oriT-aac(3)IV*-FRT by λ-red mediated recombination, Apra^{R}, Kan^{R}	this study
6F04-Δ*rokB*	6F04-Δ*rokB*-apra, resistance cassette removed by FLP recombinase, Kan^{R}	this study
plasmids	description	source/reference
pIJ790	λ-RED (*gam*, *bet*, *exo*), *cat*, *araC*, rep101ts	(7)
pIJ773	P1-FRT-*oriT-aac(3)IV*-FRT-P2, Apr^{R}	(7)
pUZ8002	OriT- RP4 derivative, Kan^{R}	(7)
BT340	FLP recombinase plasmid, *ts*, Carb^{R}, Cml^{R}	(7)

pGEM-T	Amp^R, f1ori, *lacZ*, linear cloning vector with T overhangs	Promega Corporation, Madison WI.
pPBB3	pGEM-T + *rokB* with restriction sites for *Eco*RI/*Bam*HI	this study
pPBB7	pGEM-T + *rokB* with restriction sites for *Hind*III/*Spe*I	this study
pUWL-apra-oriT	*Streptomyces*-*E. coli*-Shuttle vector, Carb^R, Apra^R, constitutive *ermE*p* Promotor, pIJ101-origin, ColE1-origin, oriT	(8)
pPBB12	pUWL-apra-oriT + *rokB* cloned as *Hind*III/*Spe*I fragment from pPBB7	this study
pGEX-6P-1	GST Expression Vector, PreScission cleavage site, Amp^R, *lacI*, ori	GE Healthcare, Freiburg, Germany
pPBB16	pGEX-6P-1 + *rokB* cloned as *Eco*RI/*Bam*HI fragment from pPBB3	this study

b Oligonucleotides used in this study

primer	primer sequence	description
PnovEforward	CGT CAC CGA TCT GAC TCG CG	586 bp promoter fragment upstream of *novE* (PnovE)+ 23 bp DAC Biotin = 609 bp
PnovEreverse	GAG GAG TCG TCG ATG TGG AGA CCT CCC CGC CCT TTC GAA GC	
PnovGforward	GTC GTG GGT GAA CTG GAG AAG	553 bp promoter fragment upstream of *novG* (PnovG) +23 bp DAC Biotin = 576 bp
PnovGreverse	GAG GAG TCG TCG ATG TGG AGA CCT GGT TGC CTT GAG CGA AGC C	
PnovHforward	AGC AGG CCG TGA TGA AAC TGC	542 bp promoter fragment upstream of *novH* (PnovH)+ 23 bp DAC Biotin = 565 bp
PnovHreverse	GAG TCG TCG ATG TGG AGA CCC GAT TCC GTC GGT ATT GTC GG	
PhrdBforward	GTC AAC TTC TGA CCG TCC AC	559 bp promoter fragment upstream of *hrdB* (PhrdB)+ 23 bp DAC Biotin = 582 bp
PhrdBreverse	GAG GAG TCG TCG ATG TGG AGA CC AAT GAG CGC CAT GAC AGA G	
DAC Biotin	Biotin-GAG GAG TCG TCG ATG TGG AGA CC	biotinylated DAC primer
RokBpUWLforw	AAG CTT GAT GGG GAA GTA AGC ACG TAC A	1263 bp, *rokB* flanked by *Hind*III/*Spe*I restriction sites
RokBpUWLrev	ACT AGT GGG TGT TTC AGG AAG TGG TG	
RokBfpGEX	GGA TCC GAT GGG GAA GTA AGC ACG TAC A	

RokBrpGEX	GAA TTC GGG TGT TTC AGG AAG TGG TG	1263 bp, *sco6115* flanked by *Bam*HI/*Eco*RI restriction sites
RokBKOf	CTG CAT CGG ACC GAC GAT GGG GAA GTA AGC ACG TAC ATG ATT CCG GGG ATC CGT CGA CC	Position 2624 - 3847 on cosmid 6F04, flanked by 39 bp up- und downstream, which enable λ Red/ET-mediated recombination
RrokBKOr	TGC GGG TGG CGC GGG TGG GCG TTG CTT CGG GGT GTT TCA TGT AGG CTG GAG CTG CTT C	
RokB_FTEST	GTC TGC ATC GGA CCG ACG	Test primer for verification of *rokB* deletion
RokB_RTEST	TGG CTC CTC GTT CGT GCG	
rokBGenF	ATC ACC CAC ATC CGC ATC G	Test primer binding in the gene *rokB*
rokBGenR	GCG TCC GGG TTG AAG AAG TT	
pUWLtest_F	ACG CCT GGT CGA TGT CGG AC	Test primer for pUWL-apra-oriT vector
pUWLtest_R	GAG CGA GGA AGC GGA AGA GC	

oligonucleotide	sequence
Biotinylated linker	Biotin-gcaggaggacgtagggtagg
PhrdB-NC_For	ACGGCGCTGTGCATCTCCCCGGCCCGCCCGCACCGTCGGCCCATTCCCAAGCCGGTGGTCGcct accctacgtcctcctgc
PhrdB-NC_Rev	CGACCACCGGCTTGGGAATGGGCCGACGGTGCGGGCGGGCCGGGGAGATGCACAGCGCCGT
Oligo_forw_1	AGCAGGCCGTGATGAAACTGCGCCCCGCCGCGGCTCAGCGGAGCGGCCCGGACTACGGCCccta ccctacgtcctcctgc
Oligo_rev_1	GGCCGTAGTCCGGGCCGCTCCGCTGAGCCGCGGCGGGGCGCAGTTTCATCACGGCCTGCT
Oligo_forw_2	CGGCTCAGCGGAGCGGCCCGGACTACGGCCGCGTACTGGAAAACCTGCGCAAGGACCCGTccta ccctacgtcctcctgc
Oligo_rev_2	ACGGGTCCTTGCGCAGGTTTTCCAGTACGCGGCCGTAGTCCGGGCCGCTCCGCTGAGCCG
Oligo_forw_3	GCGTACTGGAAAACCTGCGCAAGGACCCGTCGCTGCGCTTCACCGACCTCGGCCGGCGCCccta ccctacgtcctcctgc
Oligo_rev_3	GGCGCCGGCCGAGGTCGGTGAAGCGCAGCGACGGGTCCTTGCGCAGGTTTTCCAGTACGC
Oligo_forw_4	CGCTGCGCTTCACCGACCTCGGCCGGCGCCTGCTCCGCCTGCTCGACGGCTCCGTCCCCGccta ccctacgtcctcctgc
Oligo_rev_4	CGGGGACGGAGCCGTCGAGCAGGCGGAGCAGGCGCCGGCCGAGGTCGGTGAAGCGCAGCG
Oligo_forw_5	TGCTCCGCCTGCTCGACGGCTCCGTCCCCGGCAGTGTCGAGCAGATCGCCCAGATCGCGGccta ccctacgtcctcctgc
Oligo_rev_5	CCGCGATCTGGGCGATCTGCTCGACACTGCCGGGGACGGAGCCGTCGAGCAGGCGGAGCA
Oligo_forw_6	GCAGTGTCGAGCAGATCGCCCAGATCGCGGACGGCGTGCCGGAACACTGCCGGACCGTGGccta ccctacgtcctcctgc
Oligo_rev_6	CCACGGTCCGGCAGTGTTCCGGCACGCCGTCCGCGATCTGGGCGATCTGCTCGACACTGC
Oligo_forw_7	ACGGCGTGCCGGAACACTGCCGGACCGTGGTGGTCGACATGGCCCGCGAGTGCGCCGCCGccta ccctacgtcctcctgc

Oligo_rev_7	CGGCGGCGCACTCGCGGGCCATGTCGACCACCACGGTCCGGCAGTGTTCCGGCACGCCGT
Oligo_forw_8	TGGTCGACATGGCCCGCGAGTGCGCCGCCGCCTGGCAGCATCTCGCCGACCAGCTCGCCGcctaccctacgtcctcctgc
Oligo_rev_8	CGGCGAGCTGGTCGGCGAGATGCTGCCAGGCGGCGGCGCACTCGCGGGCCATGTCGACCA
Oligo_forw_9	CCTGGCAGCATCTCGCCGACCAGCTCGCCGACCGGGACACCGCCTGACGTTCAACTGCTCccctaccctacgtcctcctgc
Oligo_rev_9	GAGCAGTTGAACGTCAGGCGGTGTCCCGGTCGGCGAGCTGGTCGGCGAGATGCTGCCAGG
Oligo__forw10	ACCGGGACACCGCCTGACGTTCAACTGCTCGAATCATTCGGTTGAACACCACCGCATGGCcctaccctacgtcctcctgc
Oligo_rev_10	GCCATGCGGTGGTGTTCAACCGAATGATTCGAGCAGTTGAACGTCAGGCGGTGTCCCGGT
Oligo_forw_11	GAATCATTCGGTTGAACACCACCGCATGGCATTTGCCTCTTGAACCGTCCCGGTGCGATGcctaccctacgtcctcctgc
Oligo_rev_11	CATCGCACCGGGACGGTTCAAGAGGCAAATGCCATGCGGTGGTGTTCAACCGAATGATTC
Oligo_forw_12	ATTTGCCTCTTGAACCGTCCCGGTGCGATGCGGAGGATGTTTCCGGGGCGGTTGTCGGTGcctaccctacgtcctcctgc
Oligo_rev_12	CACCGACAACCGCCCCGGAAACATCCTCCGCATCGCACCGGGACGGTTCAAGAGGCAAAT
Oligo_forw_13	CGGAGGATGTTTCCGGGGCGGTTGTCGGTGCGAGCTGTCCCGGGAGTCGCCTTTTCGCGCcctaccctacgtcctcctgc
Oligo_rev_13	GCGCGAAAAGGCGACTCCCGGGACAGCTCGCACCGACAACCGCCCCGGAAACATCCTCCG
Oligo_forw_14	CGAGCTGTCCCGGGAGTCGCCTTTTCGCGCAGAATTCCCCTTCGGTCATCCGGGGAGTACcctaccctacgtcctcctgc
Oligo_rev_14	GTACTCCCCGGATGACCGAAGGGGAATTCTGCGCGAAAAGGCGACTCCCGGGACAGCTCG
Oligo_forw_15	AGAATTCCCCTTCGGTCATCCGGGGAGTACGTTCCAATGTCATGGAGACTTAAGGGGGAAcctaccctacgtcctcctgc
Oligo_rev_15	TTCCCCCTTAAGTCTCCATGACATTGGAACGTACTCCCCGGATGACCGAAGGGGAATTCT
Oligo_forw_16	GTTCCAATGTCATGGAGACTTAAGGGGGAAGTTTGTTCAACACACGTGCGAACAAAGCTTcctaccctacgtcctcctgc
Oligo_rev_16	AAGCTTTGTTCGCACGTGTGTTGAACAAACTTCCCCCTTAAGTCTCCATGACATTGGAAC
Oligo_forw_17	GTTTGTTCAACACACGTGCGAACAAAGCTTCTGATCAGTCGCCGACAATACCGACGGAATcctaccctacgtcctcctgc
Oligo_rev_17	ATTCCGTCGGTATTGTCGGCGACTGATCAGAAGCTTTGTTCGCACGTGTGTTGAACAAAC
Oligo_forw_18	TTGTTCAACACACGTGCGAACAAAGCTTCTGATCAGTCGCCGACAATACCGACGGAATCGcctaccctacgtcctcctgc
Oligo_rev_18	CGATTCCGTCGGTATTGTCGGCGACTGATCAGAAGCTTTGTTCGCACGTGTGTTGAACAA
PnovH-LH_F	GGGGGAAGTTTGTTCAACACACGTGCGAACAAAGCTTCTGATcctaccctacgtcctcctgc
PnovH-LH_R	ATCAGAAGCTTTGTTCGCACGTGTGTTGAACAAACTTCCCCC
PnovH-LH-2_F	GGGAAGTTTGTTCAACACACGTGCGAACAAAGCTTCTGATcctaccctacgtcctcctgc
PnovH-LH-2_R	ATCAGAAGCTTTGTTCGCACGTGTGTTGAACAAACTTCCC
PnovH-LH-4_F	GAAGTTTGTTCAACACACGTGCGAACAAAGCTTCTGATcctaccctacgtcctcctgc
PnovH-LH-4_R	ATCAGAAGCTTTGTTCGCACGTGTGTTGAACAAACTTC

PnovH-LH-6_F	AGTTTGTTCAACACACGTGCGAACAAAGCTTCTGATcctaccctacgtcctcctgc
PnovH-LH-6_R	ATCAGAAGCTTTGTTCGCACGTGTGTTGAACAAACT
PnovH-LH-8_F	TTTGTTCAACACACGTGCGAACAAAGCTTCTGATcctaccctacgtcctcctgc
PnovH-LH-8_R	ATCAGAAGCTTTGTTCGCACGTGTGTTGAACAAA
PnovH-LH-10_F	TGTTCAACACACGTGCGAACAAAGCTTCTGATcctaccctacgtcctcctgc
PnovH-LH-10_R	ATCAGAAGCTTTGTTCGCACGTGTGTTGAACA
PnovH-LH-12_F	TTCAACACACGTGCGAACAAAGCTTCTGATcctaccctacgtcctcctgc
PnovH-LH-12_R	ATCAGAAGCTTTGTTCGCACGTGTGTTGAA
PnovH-LH-14_F	CAACACACGTGCGAACAAAGCTTCTGATcctaccctacgtcctcctgc
PnovH-LH-14_R	ATCAGAAGCTTTGTTCGCACGTGTGTTG
PnovH-RH_F	ATCAGAAGCTTTGTTCGCACGTGTGTTGAACAAACTTCCCCCcctaccctacgtcctcctgc
PnovH-RH_R	GGGGGAAGTTTGTTCAACACACGTGCGAACAAAGCTTCTGAT
PnovH-RH-2_F	CAGAAGCTTTGTTCGCACGTGTGTTGAACAAACTTCCCCCcctaccctacgtcctcctgc
PnovH-RH-2_R	GGGGGAAGTTTGTTCAACACACGTGCGAACAAAGCTTCTG
PnovH-RH-4_F	GAAGCTTTGTTCGCACGTGTGTTGAACAAACTTCCCCCcctaccctacgtcctcctgc
PnovH-RH-4_R	GGGGGAAGTTTGTTCAACACACGTGCGAACAAAGCTTC
PnovH-RH-6_F	AGCTTTGTTCGCACGTGTGTTGAACAAACTTCCCCCcctaccctacgtcctcctgc
PnovH-RH-6_R	GGGGGAAGTTTGTTCAACACACGTGCGAACAAAGCT
PnovH-RH-8_F	CTTTGTTCGCACGTGTGTTGAACAAACTTCCCCCcctaccctacgtcctcctgc
PnovH-RH-8_R	GGGGGAAGTTTGTTCAACACACGTGCGAACAAAG
PnovH-RH-10_F	TTGTTCGCACGTGTGTTGAACAAACTTCCCCCcctaccctacgtcctcctgc
PnovH-RH-10_R	GGGGGAAGTTTGTTCAACACACGTGCGAACAA
PnovH-RH-12_F	GTTCGCACGTGTGTTGAACAAACTTCCCCCcctaccctacgtcctcctgc
PnovH-RH-12_R	GGGGGAAGTTTGTTCAACACACGTGCGAAC
PnovH-RH-14_F	TCGCACGTGTGTTGAACAAACTTCCCCCcctaccctacgtcctcctgc
PnovH-RH-14_R	GGGGGAAGTTTGTTCAACACACGTGCGA
PnovHbind_F	AGTTTGTTCAACACACGTGCGAACAAAGcctaccctacgtcctcctgc
PnovHbind_R	CTTTGTTCGCACGTGTGTTGAACAAACT
ProkB/sco6114-BS1F 40bp	GGCGTACTTATTTCATTCATGTACGTGCTTACTTCCCCATcctaccctacgtcctcctgc
ProkB/sco6114-BS1R 40bp	ATGGGGAAGTAAGCACGTACATGAATGAAATAAGTACGCC
ProkB/sco6114-BS2F 40bp	GTCCGGACTTTTTTCAGAAACGGAGATACTTAGTTGTTACcctaccctacgtcctcctgc
ProkB/sco6114-BS2R 40bp	GTAACAACTAAGTATCTCCGTTTCTGAAAAAAGTCCGGAC
ProkB/sco6114-BS1_RHF	GTACTTATTTCATTCATGTACGTGCTTACTTCCCCATcctaccctacgtcctcctgc
ProkB/sco6114-BS1_RHR	ATGGGGAAGTAAGCACGTACATGAATGAAATAAGTAC

ProkB/sco6114-BS1_RHF-2	ACTTATTTCATTCATGTACGTGCTTACTTCCCCATcctaccctacgtcctcctgc
ProkB/sco6114-BS1_RHR-2	ATGGGGAAGTAAGCACGTACATGAATGAAATAAGT
ProkB/sco6114-BS1_RHF-4	CTTATTTCATTCATGTACGTGCTTACTTCCCCATcctaccctacgtcctcctgc
ProkB/sco6114-BS1_RHR-4	ATGGGGAAGTAAGCACGTACATGAATGAAATAAG
ProkB/sco6114-BS1_RHF-6	TATTTCATTCATGTACGTGCTTACTTCCCCATcctaccctacgtcctcctgc
ProkB/sco6114-BS1_RHR-6	ATGGGGAAGTAAGCACGTACATGAATGAAATA
ProkB/sco6114-BS1_RHF-8	TTTCATTCATGTACGTGCTTACTTCCCCATcctaccctacgtcctcctgc
ProkB/sco6114-BS1_RHR-8	ATGGGGAAGTAAGCACGTACATGAATGAAA
ProkB/sco6114-BS1_RHF-10	TCATTCATGTACGTGCTTACTTCCCCATcctaccctacgtcctcctgc
ProkB/sco6114-BS1_RHR-10	ATGGGGAAGTAAGCACGTACATGAATGA
ProkB/sco6114-BS1_LHF	GGAAGTAAGCACGTACATGAATGAAATAAGTACGCCcctaccctacgtcctcctgc
ProkB/sco6114-BS1_LHR	GGCGTACTTATTTCATTCATGTACGTGCTTACTTCC
ProkB/sco6114-BS1_LHF-2	AAGTAAGCACGTACATGAATGAAATAAGTACGCCcctaccctacgtcctcctgc
ProkB/sco6114-BS1_LHR-2	GGCGTACTTATTTCATTCATGTACGTGCTTACTT
ProkB/sco6114-BS1_LHF-4	GTAAGCACGTACATGAATGAAATAAGTACGCCcctaccctacgtcctcctgc
ProkB/sco6114-BS1_LHR-4	GGCGTACTTATTTCATTCATGTACGTGCTTAC
ProkB/sco6114-BS1_LHF-6	AAGCACGTACATGAATGAAATAAGTACGCCcctaccctacgtcctcctgc
ProkB/sco6114-BS1_LHR-6	GGCGTACTTATTTCATTCATGTACGTGCTT
ProkB/sco6114-BS1_LHF-8	GCACGTACATGAATGAAATAAGTACGCCcctaccctacgtcctcctgc
ProkB/sco6114-BS1_LHR-8	GGCGTACTTATTTCATTCATGTACGTGC

ProkB/sco6114-BS2_RHF	GGACTTTTTTCAGAAACGGAGATACTTAGTTGTTACcctaccctacgtcctcctgc
ProkB/sco6114-BS2_RHR	GTAACAACTAAGTATCTCCGTTTCTGAAAAAAGTCC
ProkB/sco6114-BS2_RHF-2	ACTTTTTTCAGAAACGGAGATACTTAGTTGTTACcctaccctacgtcctcctgc
ProkB/sco6114-BS2_RHR-2	GTAACAACTAAGTATCTCCGTTTCTGAAAAAAGT
ProkB/sco6114-BS2_RHF-4	TTTTTTCAGAAACGGAGATACTTAGTTGTTACcctaccctacgtcctcctgc
ProkB/sco6114-BS2_RHR-4	GTAACAACTAAGTATCTCCGTTTCTGAAAAAA
ProkB/sco6114-BS2_RHF-6	TTTTCAGAAACGGAGATACTTAGTTGTTACcctaccctacgtcctcctgc
ProkB/sco6114-BS2_RHR-6	GTAACAACTAAGTATCTCCGTTTCTGAAAA
ProkB/sco6114-BS2_RHF-8	TTCAGAAACGGAGATACTTAGTTGTTACcctaccctacgtcctcctgc
ProkB/sco6114-BS2_RHR-8	GTAACAACTAAGTATCTCCGTTTCTGAA
ProkB/sco6114-BS2_LHF	AACAACTAAGTATCTCCGTTTCTGAAAAAAGTCCGGACcctaccctacgtcctcctgc
ProkB/sco6114-BS2_LHR	GTCCGGACTTTTTTCAGAAACGGAGATACTTAGTTGTT
ProkB/sco6114-BS2_LHF-2	CAACTAAGTATCTCCGTTTCTGAAAAAAGTCCGGACcctaccctacgtcctcctgc
ProkB/sco6114-BS2_LHR-2	GTCCGGACTTTTTTCAGAAACGGAGATACTTAGTTG
ProkB/sco6114-BS2_LHF-4	ACTAAGTATCTCCGTTTCTGAAAAAAGTCCGGACcctaccctacgtcctcctgc
ProkB/sco6114-BS2_LHR-4	GTCCGGACTTTTTTCAGAAACGGAGATACTTAGT
ProkB/sco6114-BS2_LHF-6	TAAGTATCTCCGTTTCTGAAAAAAGTCCGGACcctaccctacgtcctcctgc
ProkB/sco6114-BS2_LHR-6	GTCCGGACTTTTTTCAGAAACGGAGATACTTA
ProkB/sco6114-BS2_LHF-8	AGTATCTCCGTTTCTGAAAAAAGTCCGGACcctaccctacgtcctcctgc
ProkB/sco6114-BS2_LHR-8	GTCCGGACTTTTTTCAGAAACGGAGATACT

ProkB/sco6114-BS1F	ACTTATTTCATTCATGTACGTGCTTACTTCCcctaccctacgtcctcctgc
ProkB/sco6114-BS1R	GGAAGTAAGCACGTACATGAATGAAATAAGT
ProkB/sco6114-BS2F	ACTTTTTTCAGAAACGGAGATACTTAGTTGcctaccctacgtcctcctgc
ProkB/sco6114-BS2R	CAACTAAGTATCTCCGTTTCTGAAAAAAGT
Psco1440-BS_F	ACGTGTCTCCTCGTCGTACGTGCTTACTGGcctaccctacgtcctcctgc
Psco1440-BS_R	CCAGTAAGCACGTACGACGAGGAGACACGT
Psco6108-BS_F	GAAGAAGGGAAGTACGTTGCTCAACAAAGGcctaccctacgtcctcctgc
Psco6108-BS_R	CCTTTGTTGAGCAACGTACTTCCCTTCTTC
Psco0812-BS_F	AATCGTTTCACACATGGTCGTACTCACAGCcctaccctacgtcctcctgc
Psco0812-BS_R	GCTGTGAGTACGACCATGTGTGAAACGATT
Psco5835-BS_F	CAATTCCGCACGCACGGGTGAGAAAGAGGTcctaccctacgtcctcctgc
Psco5835-BS_R	ACCTCTTTCTCACCCGTGCGTGCGGAATTG
Psco2183-BS_F	ACGAGTTACAGTCACAGACGGGCTTCCACCcctaccctacgtcctcctgc
Psco2183-BS_R	GGTGGAAGCCCGTCTGTGACTGTAACTCGT
Psco3206-BS_F	GGCTGTTTGAGACATGGGGATACCCACTTCcctaccctacgtcctcctgc
Psco3206-BS_R	GAAGTGGGTATCCCCATGTCTCAAACAGCC
Psco0938-BS_F	CGTGACTTCCGTCACGGACGTGTGTGCGTCcctaccctacgtcctcctgc
Psco0938-BS_R	GACGCACACACGTCCGTGACGGAAGTCACG
Psco2924-BS_F	CCTCTCCCGAGTCTCGTACGTCCTTGCGTGcctaccctacgtcctcctgc
Psco2924-BS_R	CACGCAAGGACGTACGAGACTCGGGAGAGG
Psco6554-BS_F	CCTCTCTCCCGTGTCGTACGTCTTTGCGTGcctaccctacgtcctcctgc
Psco6554-BS_R	CACGCAAAGACGTACGACACGGGAGAGAGG
Psco3989-BS_F	GCTGTCTCCCGCAACGTACGAGCGCCCTTCcctaccctacgtcctcctgc
Psco3989-BS_R	GAAGGGCGCTCGTACGTTGCGGGAGACAGC
Psco1403-BS_F	CGTGTCCTCGGACACGGACGAGCGGTCTGCcctaccctacgtcctcctgc
Psco1403-BS_R	GCAGACCGCTCGTCCGTGTCCGAGGACACG
Psco3678-BS_F	CGCGTCTTGCGTATCCTCGGTGCGTGCTTCcctaccctacgtcctcctgc
Psco3678-BS_R	GAAGCACGCACCGAGGATACGCAAGACGCG
Psco3215-BS_F	CCTTACTTTCGACAGGGACGGATCTTGGCCcctaccctacgtcctcctgc
Psco3215-BS_R	GGCCAAGATCCGTCCCTGTCGAAAGTAAGG
Psco2446-BS_F	CCTGTGCTCATCCACGTCTCTCTTCGTGTCcctaccctacgtcctcctgc
Psco2446-BS_R	GACACGAAGAGAGACGTGGATGAGCACAGG

1. MacNeil DJ, Gewain KM, Ruby CL, Dezeny G, Gibbons PH, MacNeil T. Analysis of *Streptomyces avermitilis* genes required for avermectin biosynthesis utilizing a novel integration vector. Gene. 1992;111(1):61-8.
2. Datsenko KA, Wanner BL. One-step inactivation of chromosomal genes in *Escherichia coli* K-12 using PCR products. Proc Natl Acad Sci U S A. 2000;97(12):6640-5.
3. Floriano B, Bibb M. *afsR* is a pleiotropic but conditionally required regulatory gene for antibiotic production in *Streptomyces coelicolor* A3(2). Mol Microbiol. 1996;21(2):385-96.
4. Eustaquio AS, Gust B, Galm U, Li SM, Chater KF, Heide L. Heterologous expression of novobiocin and clorobiocin biosynthetic gene clusters. Appl Environ Microbiol. 2005;71(5):2452-9.
5. Kominek LA. Biosynthesis of novobiocin by *Streptomyces niveus*. Antimicrob Agents Chemother. 1972;1(2):123-34.
6. Redenbach M, Kieser HM, Denapaite D, Eichner A, Cullum J, Kinashi H, et al. A set of ordered cosmids and a detailed genetic and physical map for the 8 Mb *Streptomyces coelicolor* A3(2) chromosome. Mol Microbiol. 1996;21(1):77-96.
7. Gust B, Challis GL, Fowler K, Kieser T, Chater KF. PCR-targeted *Streptomyces* gene replacement identifies a protein domain needed for biosynthesis of the sesquiterpene soil odor geosmin. Proc Natl Acad Sci U S A. 2003;100(4):1541-6.
8. Hardter U. Heterologe Expression der Glykosyltransferasen SagGT1 und SagGT2 des Saquamycin Z-Produzenten *Micromonospora* sp. Tü 6368. In: Freiburg A-LU, editor. Diplomarbeit Albert-Ludwigs Universität Freiburg2007.
